# One Health implications and first evidence of environmental contamination of helminths in soil from goat farms in Ratchaburi, Thailand

**DOI:** 10.1007/s00436-025-08541-w

**Published:** 2025-08-07

**Authors:** Abigail Hui En Chan, Wallop Pakdee, Chanisara Kaenkaew, Sivapong Sungpradit, Vachirapong Charoennitiwat, Teera Kusolsuk, Urusa Thaenkham

**Affiliations:** 1 Laboratory of Helminth Biodiversity and Drug Development, Department of Helminthology, Faculty of Tropical Medicine, Mahidol University, Bangkok, Thailand; 2 Department of Pre-Clinic and Applied Animal Science, Faculty of Veterinary Science, Mahidol University, Nakhon Pathom, Thailand

**Keywords:** Goat, Zoonotic helminths, Soil, One Health, Thailand

## Abstract

Zoonotic helminths are responsible for the majority of helminthic infections occurring in humans globally. Environmental systems serve as a reservoir for zoonotic helminths, facilitating their transmission to humans and animals. Livestock farms may serve as hotspots for zoonotic transmission, increasing infection risk. Focusing on goat farms in Ratchaburi Province, Thailand, we aim to detect and identify zoonotic helminths present in the soil environment through morphological and molecular techniques. Soil samples (*n* = 270) were collected from 30 goat farms in Ratchaburi Province, and helminths were morphologically identified using a modified sedimentation and flotation method. Helminths were then molecularly identified using the nuclear 18S rRNA gene and the internal transcribed spacer 2 region. The helminths identified include human, livestock-parasitic nematodes, trematodes, cestodes, plant-parasitic nematodes, insect-parasitic nematodes, and free-living nematodes. Morphological and molecular detection show that 80% and 86% of the farms were positive for helminths, respectively. From 30 farms, 60% were positive for livestock- or human-parasitic helminths, with eight species detected. Moreover, 50% of farms were positive for either *Haemonchus contortus* or *Trichostrongylus colubriformis*. Utilizing soil as a non-invasive method for the detection and identification of helminths contaminated in the soil, this study demonstrated the presence of human- and animal-parasitic helminths with the soil matrix serving as a shared environment and reservoir for zoonotic transmission of helminthic infection, further emphasizing the importance of the One Health approach towards mitigating parasitic disease transmission. The findings showed significant implications for the incorporation of soil-based methods into sustainable helminth infection control programs.

**Supplementary information:**

The online version contains supplementary material available at 10.1007/s00436-025-08541-w.

## Introduction

Zoonotic helminths, which infect both humans and animals, are responsible for the majority of helminthic infections occurring in humans globally (Robinson & Dalton 2009). These include food-borne nematodes, trematodes, cestodes, and gastrointestinal helminths. Climatic changes, increased anthropogenic activities, reduced interface between humans and animals, and changed dietary lifestyles are factors that facilitate the increase in spillover events and consequent zoonoses occurring (Esposito et al. 2023; Rupasinghe et al. 2022). Additionally, over 70% of helminth species have high potential to result in zoonoses (Majewska et al. 2021). Environmental systems such as soil serve as a reservoir for zoonotic helminths, facilitating their transmission to both humans and animals (Amoah et al. 2017). As helminth eggs are excreted via the feces of definitive hosts into the soil environment, they can persist and further develop into infective stages before completing the life cycle through either direct contact or ingestion by the animal or human host (Gordon et al. 2017).

Livestock farms, including goat farms, may serve as hotspots for the transmission of zoonotic helminths, where helminth eggs can easily contaminate the soil through animal feces (Gurmassa et al. 2023). The soil environment serves as a critical link in the transmission cycle of zoonotic helminths, facilitating their spread from animal to animal, animal to human, and vice versa (Penakalapati et al. 2017). Gastrointestinal nematodes (GIN) belonging to the strongylid group are the major group of helminths infecting goats (Maurizio et al. 2023). Genera such as *Haemonchus*, *Trichostrongylus*, and *Oesophagostomum* are common GINs in goats, with *Haemonchus contortus* infection resulting in significant economic losses in the livestock industry (Arsenopoulos et al. 2021; Rodriguez et al. 2015). In Thailand, high prevalences of GIN infections in goats have been reported from various provinces including Kanchanaburi, Khon Kaen, Satun, Nakhon Pathom, and Ratchaburi, demonstrating their ubiquitous distribution across the country (Income et al. 2021; Kaewnoi et al. 2024; Ratanapob et al. 2012; Rerkyusuke et al. 2024). Importantly, sporadic human infection due to *Trichostrongylus* has been reported in Thailand, where close contact with livestock animals is a risk factor for infection (Phosuk et al. 2013).

As goats and other livestock animals (e.g., cattle, chicken, and pigs) are often allowed to graze and roam freely on farms, there is a high propensity of the soil being contaminated with helminth eggs via animal feces (Paliy et al. 2018). Evidence of the soil environment as a reservoir for helminths has been documented where zoonotic soil-transmitted helminths such as *Toxocara* and hookworms were identified. These soils were sampled from urban and rural areas in Malaysia where stray dogs and cats can be seen roaming in close contact with humans (Azian et al. 2008; Tun et al. 2015). A study conducted in Nigeria, where soil samples were collected from areas with free-range livestock, revealed a high percentage of samples (55%) positive for helminth eggs (Omudu and Amuta 2007). Soil samples from public areas in Thailand also revealed contamination with helminths such as *Toxocara*, hookworms, *Ascaris*, *Strongyloides*, *Trichuris*, and *Taenia*, indicative of the zoonotic risk with the soil environment as an avenue for transmission (Dokmaikaw and Suntaravitun 2023; Phasuk et al. 2020; Pinyopanuwat et al. 2018). However, no study has yet been conducted in Thailand to investigate helminth contamination in soil on livestock farms. Thus, the proximity of humans and animals on livestock farms may increase the risk of zoonotic helminth infections, with the soil environment providing a suitable ecosystem for helminth contamination and potential One Health impact.

Focusing on goat farms in Ratchaburi Province, Thailand, where GIN infection in goats has been reported (Junsiri et al. 2021), we aim to detect and identify zoonotic helminths present in the soil environment using morphological and molecular techniques. With the soil serving as a reservoir for helminths, we present the first evidence of helminth contamination in soil from livestock farms in Thailand. This non-invasive method offers a viable alternative for monitoring helminth infections in animals. We seek to provide insights into the zoonotic and One Health implications, which are important for the well-being of humans, animals, and the environment.

## Methods

### Goat farms and soil collection

**Fig. 1:**
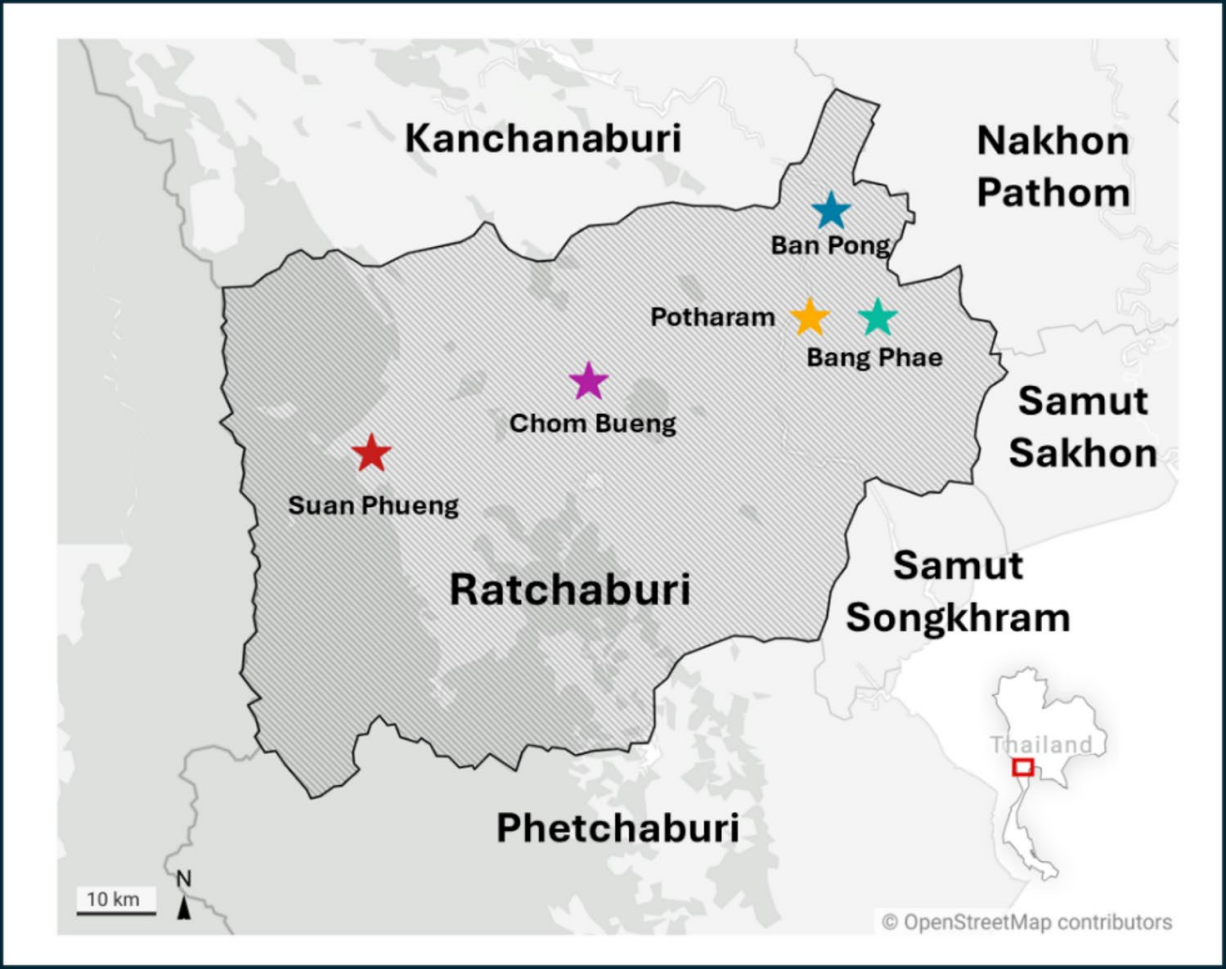
Map showing the districts where soils from goat farms were obtained

Soil samples were collected from 30 goat farms located in five districts (Chom Bueng, Suan Phueng, Potharam, Ban Pong, and Bang Phae) in Ratchaburi Province from December 2023 to June 2024. The farms were randomly selected, with six farms chosen per district. At each farm, nine soil samples were obtained, resulting in a total of 270 samples. Approximately 50 g of topsoil (top 5 cm depth) was collected using a shovel and kept in individual ziplock bags for storage and subsequent analysis. The topsoil was specifically targeted due to its higher likelihood of helminth contamination and its significant role in helminth transmission. The soil samples were maintained at a low temperature while being transported to the Department of Helminthology, Faculty of Tropical Medicine, Mahidol University, Bangkok. Figure 1 presents the districts where sampling was conducted.

### Helminth isolation and morphological identification

Helminths were isolated from soil samples using a modified sedimentation and flotation technique. Large particles such as rocks, sticks, and leaves were manually removed, and the soil was passed through a 0.2-mm sieve to remove big particles. Twenty grams of the sieved soil was weighed out and transferred to a 50-ml Falcon tube containing 1% Tween 80 solution. The remaining soil was kept in 70% ethanol and stored at − 20 °C for downstream molecular identification. The soil slurry in the Falcon tube was mixed thoroughly and allowed to sediment for 1 h to allow the dissociation of helminth eggs from soil particles. The process was repeated twice, and the supernatant was removed and replaced with water to wash the sample. Sedimentation with water was repeated twice to ensure that the 1% Tween 80 solution was washed off. After sedimentation, 30 ml of saturated sodium chloride (NaCl) was added to the sediment, and the homogenate was separated into 15-ml Falcon tubes. Additional NaCl was added to fill each Falcon tube to form a reverse meniscus at the top. A coverslip was carefully placed over the top of the Falcon tube and allowed to stand for 15 min. After 15 min, the coverslip was removed and placed on a microscope slide, where it was examined for the presence of helminth eggs under a light microscope.

Statistical analysis to compare significant differences in the percentage of helminth-positive samples was conducted using R Studio v. 1.2.5033 (R Studio Team 2020), with a predetermined significance level set at *P* < 0.05.

### Molecular identification of helminths

Prior to DNA extraction, the soil samples were washed in water to remove any ethanol that was present. Genomic DNA was then extracted from each sample using the DNeasy® PowerSoil® Pro Kit (Qiagen, Hilden, Germany) following the manufacturer’s recommendations.

Following DNA extraction, four PCR sets were conducted for each sample. The four sets are (1) 18S ribosomal RNA (rRNA) gene for nematodes (Holterman et al. 2006), (2) 18S rRNA gene for platyhelminths (Routtu et al. 2014), (3) internal transcribed spacer 2 (ITS2) region specific for *H. contortus*, and (4) ITS2 region specific for *T. colubriformis*. The 18S rRNA gene was selected as a genetic marker to allow for the broad-range detection of nematodes and platyhelminths present in the soil, while the ITS2 region was used for species-specific detection of *H. contortus* and *T. colubriformis*. The primers and thermocycling conditions for the 18S rRNA gene for nematodes followed Holterman et al. (2006), while the primers for platyhelminths followed Routtu et al. (2014). For the ITS2 species-specific primers, they were newly designed in this study for amplification of *H. contortus* and *T. colubriformis* gDNA. Briefly, reference ITS2 sequences of *Haemonchus* and *Trichostrongylus* were obtained from the NCBI database and aligned using ClustalX 2.1 and BioEdit 7.0 (Hall 1999; Thompson et al. 2002). Primers were then manually designed, and their properties and specificity were checked with OligoCalc version 3.27 (Kibbe 2007) and FastPCR (Kalendar et al. 2017). The species-specific ITS2 primers are Haem567F: 5′-GTCAAATGGCATTTGTCT-3′ and Haem684R: 5′-CAAATAGTGGCAACATGTTC-3′ for *H. contortus* and Tricho560F: 5′-AACTCTAACACTGTTTGTCG-3′ and Tricho728R: 5′-CATGTCCCTGTTTAAATCA-3′ for *T. colubriformis*.

PCR amplification was conducted separately for each genetic marker in a T100™ thermocycler (Bio-Rad, CA, USA) with a final volume of 20 µl containing 10 µl of 2X i-Taq™ mastermix (iNtRON Biotechnology, Gyeonggi, South Korea) with 10 µM of each primer. The PCR thermocycling profiles for the 18S rRNA gene followed the procedures by Holterman et al. (2006) and Routtu et al. (2014), while for the ITS2 region, the reactions used 95 °C for 5 min of initial denaturation, 35 cycles of 95 °C for 30 s, 51 °C for 30 s for *H. contortus* and 50 °C for 30 s for *T. colubriformis*, 72 °C for 30 s, followed by a final extension step at 72 °C for 10 min. Amplicons were checked and visualized on a 1% agarose gel stained with SYBR Safe (Invitrogen, MA, USA). The amplification with the 18S rRNA gene for nematodes and platyhelminths yielded amplicon sizes of 800 bp, while the ITS2 region for *H. contortus *and* T. colubriformis* yielded amplicon sizes of 110 bp and 180 bp, respectively. All positive amplicons for the 18S rRNA gene and representative amplicons for the ITS2 region were sent for FastNGS performed by a commercial company (Biotech, Beijing, China).

Electropherograms of the sequences were checked using Bioedit 7.0 (Hall 1999), followed by multiple sequence alignment using ClustalX 2.1 (Thompson et al. 2002). The aligned sequences were checked, and phylogenetic analysis using the neighbor-joining (NJ) and maximum likelihood (ML) method was conducted in MEGA X (Kumar et al. 2018). The best-fit nucleotide substitution model was used for the ML method, and 1000 bootstrap replicates were selected for tree topology support. The phylogenies were visualized and labeled with FigTree 1.3.1 (Rambaut 2010). The sequences used for phylogenetic analysis are in Supplementary Table 1.

## Results

### Morphological identification of helminths in soil

**Fig. 2:**
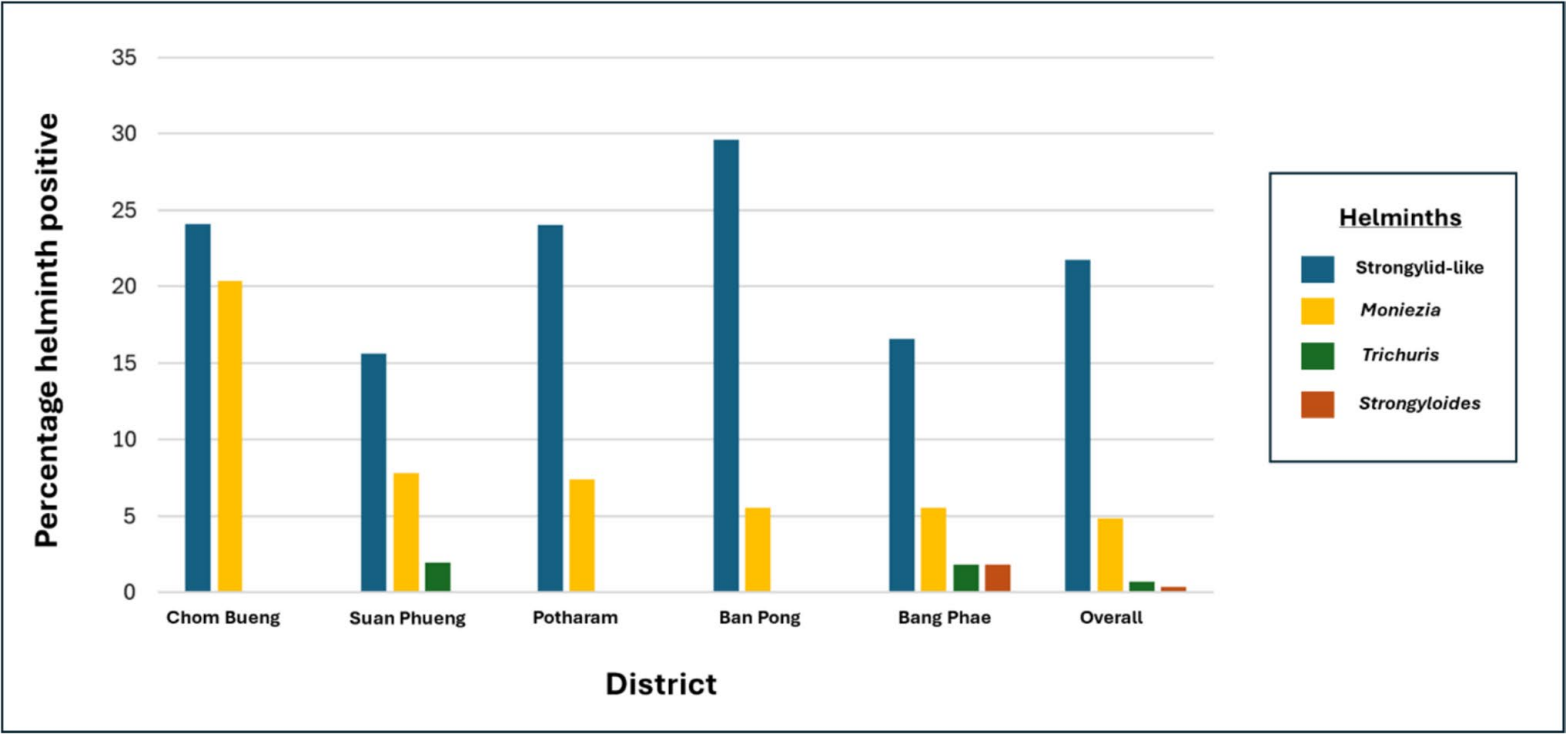
Percentage of soil samples positive for helminths per district

Of the 270 soil samples collected, 34% tested positive for the presence of helminth eggs. Helminth eggs were detected in 24 out of 30 farms (80%), with strongylid-like, *Strongyloides*, *Trichuris*, and *Moniezia* eggs identified in the soil samples. Of the four types of helminths identified, strongylid-like eggs were the majority, comprising 21% overall. Across the districts, farms in Chom Bueng had the highest number of positive samples for helminths (46%), while farms in Ban Pong had the highest percentage of samples positive for strongylid-like eggs (29%). No statistically significant difference was observed between the districts for the percentage of helminth-positive samples and for each type of helminth. Strongylid-like and *Moniezia* eggs were found in all districts, while *Trichuris* eggs were detected in Suan Phueng and Bang Phae districts. *Strongyloides* were only found in Bang Phae. Figure 2 presents the percentage of each type of helminth present in the soil samples per district. Representative images of helminth eggs obtained from the samples are depicted in Supplementary file 2.

### Molecular identification

Molecular detection of nematodes and platyhelminths in soil samples collected from the 30 farms revealed that 86% of these farms were positive for helminths. The identified helminth types include human- and livestock-parasitic nematodes, trematodes, and cestodes; plant-parasitic nematodes; insect-parasitic nematodes; and free-living nematodes. Moreover, 60% of the farms were positive for livestock- or human-parasitic helminths, with a total of eight species detected. Table 1 presents the molecular identities of the helminths found on each farm.

**Table 1:** Helminths detected from soil obtained from goat farms in Ratchaburi Province

District	Farm	Human- and livestock-parasitic nematodes	Human- and livestock-parasitic platyhelminths	Insect- and plant-parasitic and free-living nematodes
Chom Bueng	1	NA	NA	*Filenchus* sp. *Mesodorylaimus* sp.
	2	NA	NA	*Halicephalobus* sp.
	3	NA	*Fasciola gigantica*	*Halicephalobus* sp.
	4	NA	NA	NA
	5	*H. contortus* *T. colubriformis* *Trichuris* sp.	*Davaineidae* sp.	*Protorhabditis* sp. *Halicephalobus* sp.
	6	*H. contortus* *T. colubriformis*	NA	*Ditylenchus* sp.
Suan Phueng	1	NA	NA	NA
	2	*H. contortus* *T. colubriformis*	NA	NA
	3	NA	NA	*Mesodorylaimus* sp. *Howardula* sp.
	4	*H. contortus* *T. colubriformis*	NA	*Diploscapter* sp.
	5	*H. contortus* *T. colubriformis*	NA	NA
	6	*H. contortus*	NA	*Howardula* sp.
Potharam	1	*H. contortus* *T. colubriformis* *Trichuris* sp.	NA	*Mesodorylaimus* sp. *Pseudacrobeles* sp. *Diploscapter* sp.
	2	*H. contortus* *T. colubriformis* *Ascaridia* sp.	NA	*Diplogastrellus* sp. *Mesodorylaimus* sp.
	3	*H. contortus*	NA	*Halicephalobus* sp. *Diploscapter* sp. *Mesodorylaimus* sp.
	4	*T. colubriformis*	NA	*Diploscapter* sp.
	5	NA	NA	*Cephalobus* sp. *Mesodorylaimus* sp.
	6	NA	NA	*Diploscapter* sp. *Halicephalobus* sp. *Pseudacrobeles* sp.
Ban Pong	1	NA	*Gastrothylacidae* sp.	*Halicephalobus* sp. *Monochoides* sp.
	2	NA	NA	*Rhabditis* sp. *Mesodorylaimus* sp.
	3	*H. contortus* *T. colubriformis*	NA	*Mesodorylaimus* sp.
	4	*H. contortus*	NA	*Howardula* sp.
	5	*Oesophagostomum* sp.	*Gastrothylacidae* sp.	*Diplogastrellus* sp.
	6	NA	NA	*Cephalobus* sp. *Parasitorhabitis* sp. *Mesodorylaimus* sp.
Bang Phae	1	*T. colubriformis*	NA	*Protorhabditis* sp.
	2	NA	NA	NA
	3	*H. contortus* *T. colubriformis*	NA	*Halicephalobus* sp. *Howardula* sp.
	4	NA	NA	*Halicephalobus* sp. *Mesodorylaimus* sp.
	5	*H. contortus*	NA	*Halicephalobus* sp. *Diploscapter* sp. *Oigolaimella* sp.
	6	NA	NA	*Diploscapter* sp. *Protorhabditis* sp. *Rhabditis* sp. *Halicephalobus* sp.

*NA* indicates no helminth detected

**Fig. 3:**
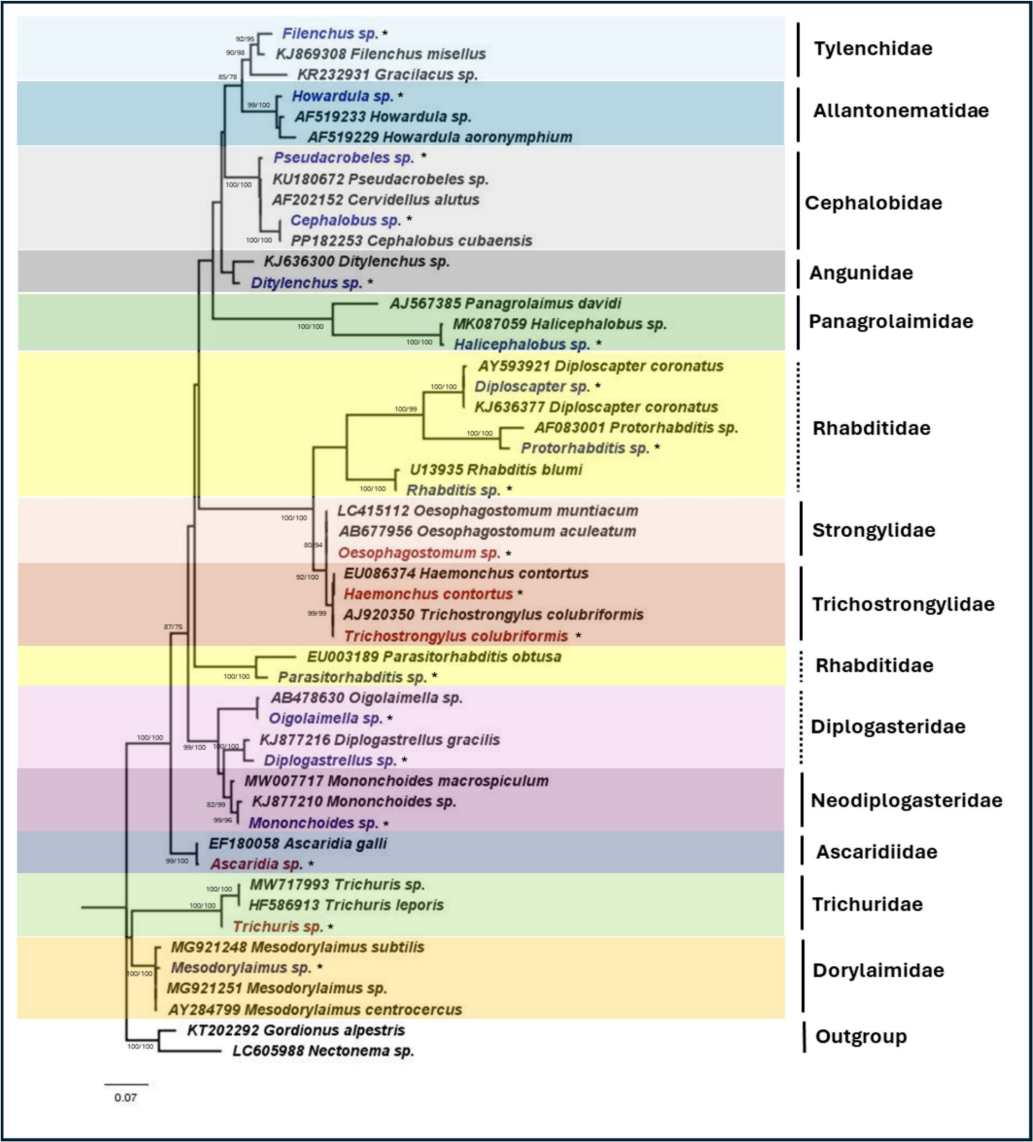
Maximum likelihood phylogeny for nematodes using the nuclear 18S rRNA gene (T3 + G). Numbers at nodes indicate bootstrap values (ML/NJ). Only bootstrap values of > 70 are shown. Representative sequences from this study are indicated with an asterisk, “*”. The human- or livestock-parasitic nematodes are indicated in “red” text

**Fig. 4:**
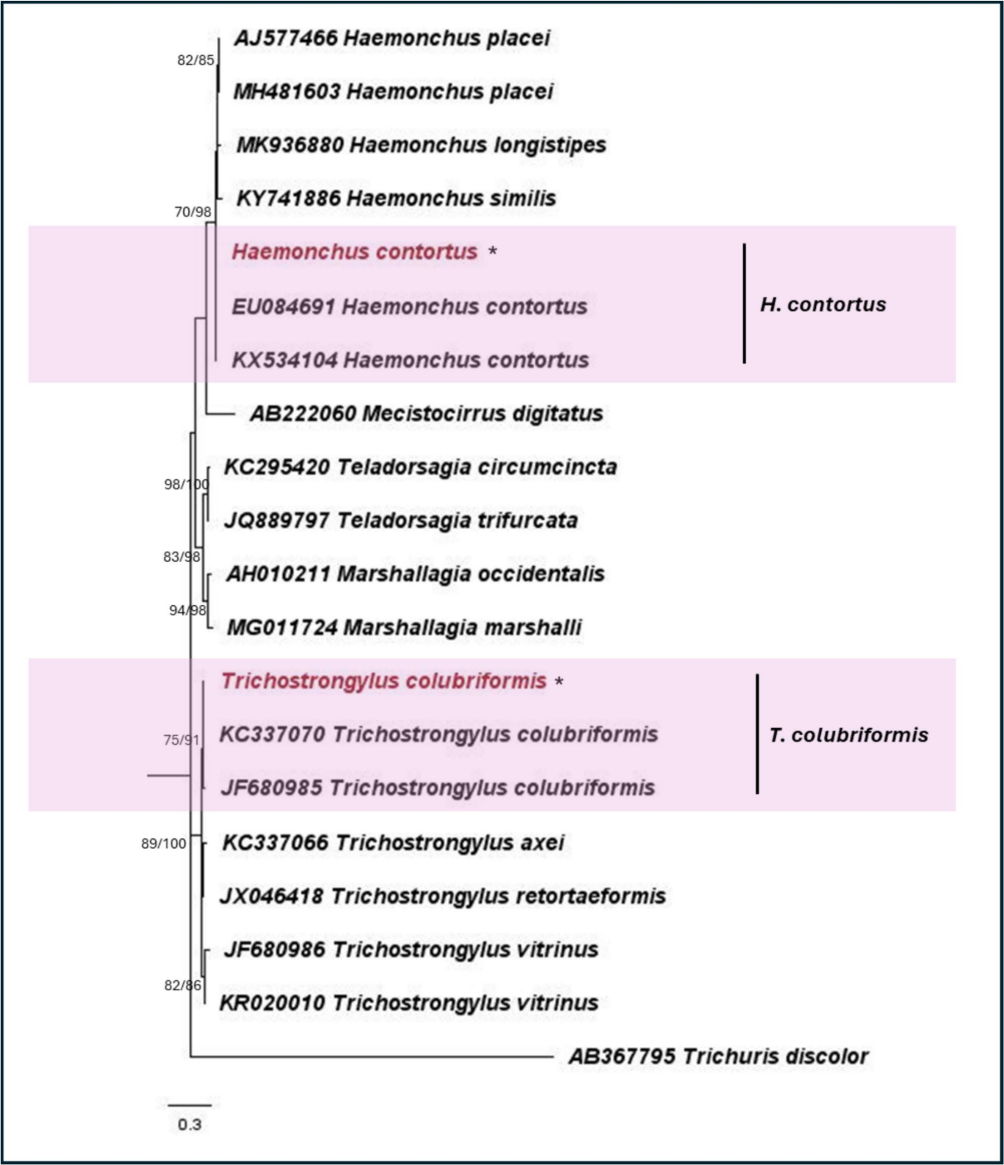
Maximum likelihood phylogeny for Trichostrongylidae using the nuclear ITS2 region (K2). Numbers at nodes indicate bootstrap values (ML/NJ). Only bootstrap values of > 70 are shown. Representative sequences from this study are indicated with an “*”

For nematodes, 19 species belonging to 13 families were identified. The ML phylogeny obtained using the 18S rRNA gene is presented in Fig. 3. Of these 19 species, five were human or animal parasitic, while the remaining 14 species are either parasitic to insects and plants or exist as free-living nematodes. The five species of human- and livestock-parasitic nematodes identified are *H. contortus*, *T. colubriformis*, *Oesophagostomum* sp., *Ascaridia* sp., and *Trichuris* sp. Additionally, with the use of ITS2 species-specific primers for the detection of *H. contortus* and *T. colubriformis*, they were detected in farms from all five districts. Figure 4 presents the phylogeny obtained using the ITS2 region. Of the 30 farms, 50% were positive for either *H. contortus* or *T. colubriformis*, demonstrating evidence of parasitic helminth contamination in the soil environment. Notably, 80% of the farms in the districts of Suan Phueng and Potharam were found to be positive for either *H. contortus* or *T. colubriformis* in the soil.

**Fig. 5:**
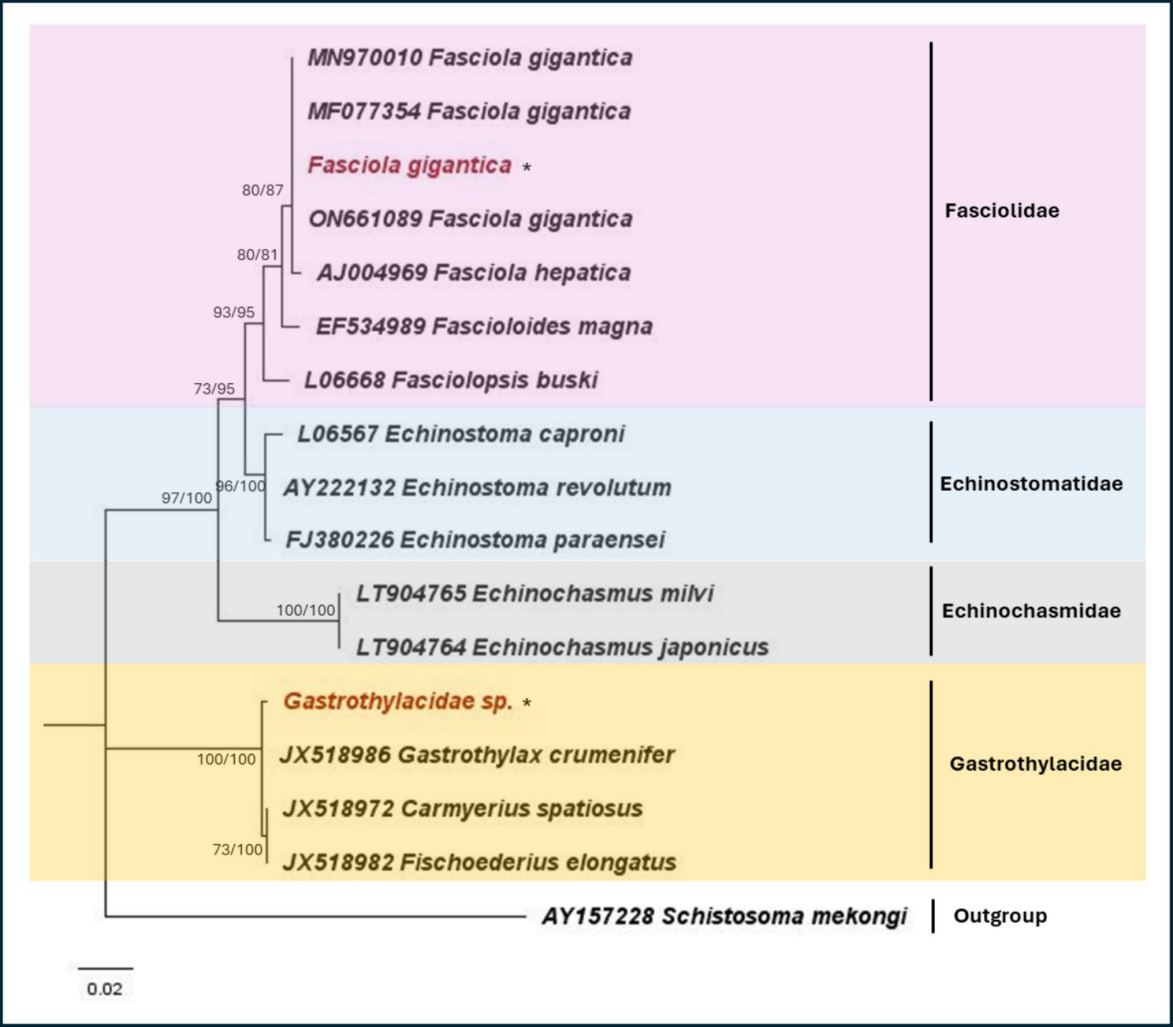
Maximum likelihood phylogeny for trematodes using the nuclear 18S rRNA gene (K2). Numbers at nodes indicate bootstrap values (ML/NJ). Only bootstrap values of > 70 are shown. Representative sequences from this study are indicated with an “*”

**Fig. 6:**
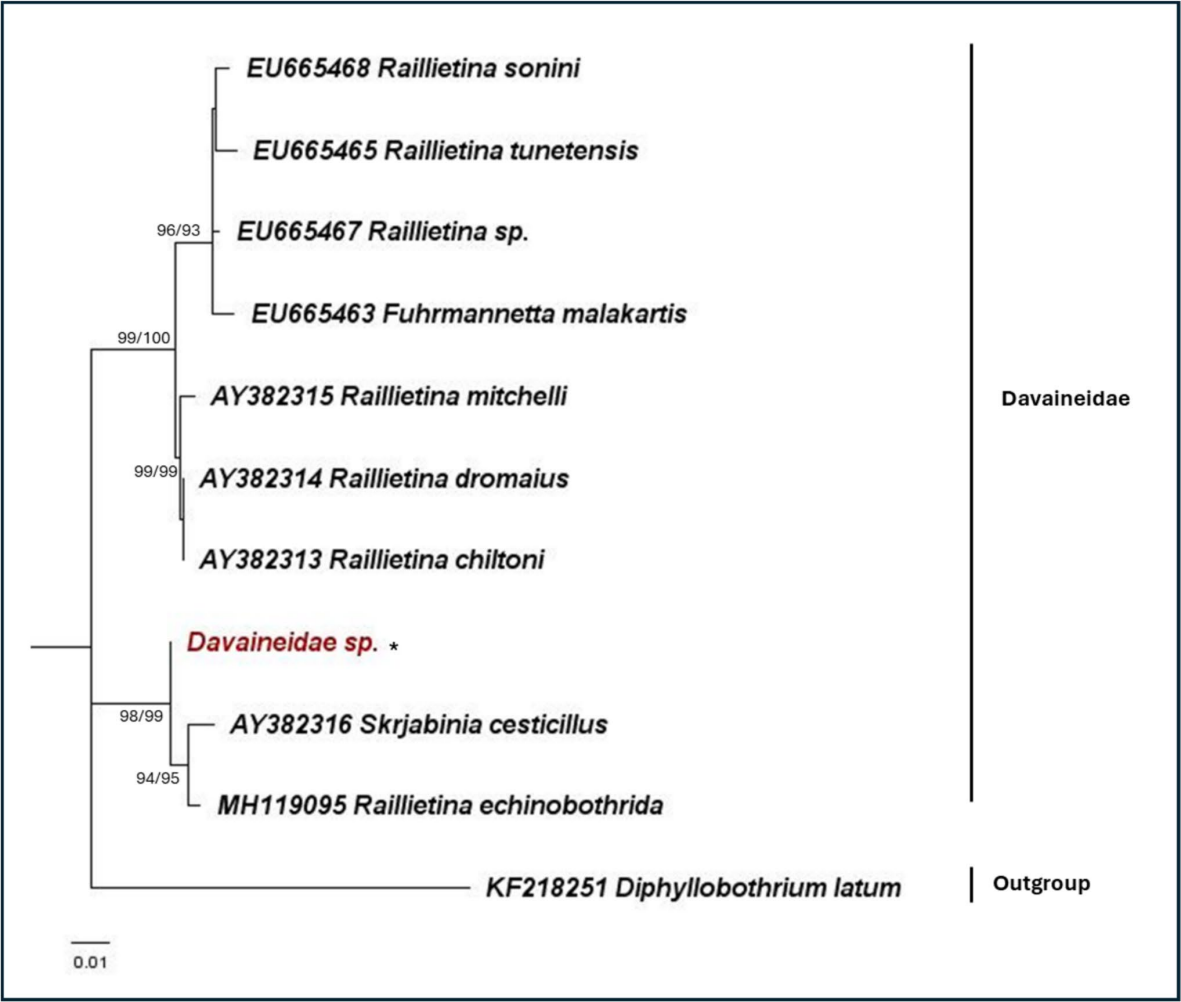
Maximum likelihood phylogeny for cestodes using the nuclear 18S rRNA gene (K2 + G). Numbers at nodes indicate bootstrap values (ML/NJ). Only bootstrap values of > 70 are shown. The representative sequence from this study is indicated with an “*”

Molecular identification of platyhelminths revealed the presence of *Fasciola gigantica*, *Gastrothylacidae* sp., and *Davaineidae* sp. in the soil samples (Figs. 5 and 6). *F. gigantica*, a human- and livestock-parasitic trematode, was detected in one farm in Chom Bueng district, while the livestock-parasitic trematode from the family Gastrothylacidae was detected in two farms in Ban Pong district. A cestode belonging to the family Davaineidae was found in one farm in Chom Bueng district.

## Discussion

Through the survey of soil samples obtained from goat farms in Ratchaburi province, helminths were successfully detected and identified using morphological and molecular methods. Most importantly, soil-based detection in the farms revealed the presence of parasitic helminths that affect human and livestock, underscoring the interconnectedness of the environment, animals, and human health, which is a core principle of the One Health approach.

Overall, molecular detection of soil obtained from a considerable proportion of farms (60%) indicated the presence of human- or livestock-parasitic helminths. Both morphological and molecular results revealed nematode and cestode species commonly infecting goats, including strongylids (*H. contortus*, *T. colubriformis*, *Oesophagostomum*), *Strongyloides*, *Trichuris*, and *Moniezia*. Molecular analysis targeting the ITS2 region revealed that *H. contortus* and *T. colubriformis* were present in 50% of the farms surveyed. These results provide evidence of helminth contamination in the soil environment due to goat feces, thus acting as a reservoir for disease transmission of zoonoses. In Thailand, GINs such as *H. contortus* and *T. colubriformis* are the predominant species infecting goats and have been detected across various regions in the country (Income et al. 2021; Kaewnoi et al. 2024; Ratanapob et al. 2012; Rerkyusuke et al. 2024). Although strongylid infections were traditionally considered to be prevalent in the southern provinces (e.g., Satun, Yala, and Narathiwat), recent surveys have indicated significant infection rates in northeast and central provinces as well (Junsiri et al. 2021; Wuthijaree et al. 2022). Traditionally, investigations into strongylid infections have relied on goat fecal samples, while our results demonstrate the feasibility of utilizing soil as a non-invasive detection approach, marking the first soil-based detection in Thailand’s livestock farms. Here, our study demonstrates the presence of *H. contortus* and *T. colubriformis* in the soil environment of goat farms, consequently increasing the risk of zoonotic transmission to humans in close contact with infected goats and contaminated environments. Sporadic human infections with *H. contortus* and *T. colubriformis* have been reported in Thailand, Lao People’s Democratic Republic, South Korea, Iran, and Australia, thus threatening human health (Boreham et al. 1995; Ghadirian and Arfaa 1973; Phosuk et al. 2013; Watthanakulpanich et al. 2013).

A recent study conducted in Ratchaburi Province has revealed a significant prevalence (87%) of strongylid infection in goats using fecal samples (Chan et al. 2025). Of them, *Haemonchus* was molecularly identified in 100% of the surveyed farms, while *Trichostrongylus* was identified in 97% of the farms assessed. This indicates an increase in strongylid infections in Ratchaburi Province, contrasting with a previous study by Junsiri et al., which reported a prevalence of 11 to 18% strongylid infections in goats (Junsiri et al. 2021). Importantly, significant levels of anthelminthic resistance to albendazole and levamisole were detected in *Haemonchus* and *Trichostrongylus* populations in Ratchaburi Province, highlighting the necessity for sustainable and effective control strategies (Chan et al. 2025). Here, through a soil-based approach, the evidence of zoonotic helminth contamination in the soil environment underscores the necessity for integrated control measures. The ability of GINs to survive in the soil increases the risk of re-infection, disease spread, and the spread of anthelminthic resistant alleles. The soil-based detection method presented in this study offers a promising alternative and non-invasive strategy for monitoring and surveying GIN infections in goat farms.

Currently, Thailand lacks definitive policies and regulations regarding appropriate goat farming practices, the import and export of goats within provinces, and the usage of anthelminthic drugs. Farming practices are farm-dependent, with owners responsible for the animals under their care. With evidence of helminth contamination in the soil environment, it is crucial to take measures that safeguard both animal and human health within the One Health framework. Examples of such measures include (1) implementing soil-based methods to detect and monitor helminth infections, (2) improving farming practices and goat housing conditions (e.g., elevated shelters or designated feeding areas) to reduce the risk of infection, (3) performing infection screening prior to the export of goats to other provinces, and (4) supervising the administration of anthelminthic treatments.

Other parasitic helminths affecting humans and livestock that rarely infect goats were also identified in the soil, indicative of helminth contamination from other sources. Examples of parasitic helminths which may originate from other animals aside from goats include *F. gigantica*, *Gastrothylacidae* sp., and *Ascaridia* sp. Of these, *F. gigantica* is pathogenic to humans, and sporadic human fascioliasis cases have been reported in Thailand (Hoang Quang et al. 2024; Srihakim and Pholpark 1991). A study conducted in Nakhon Ratchasima Province revealed that the copro-prevalences of *Fasciola* in cattle ranged from 0.99% to 11.11% while seroprevalences varied between 2.94% and 45.45% (Martviset et al. 2024). The presence of other helminths in the soil indicates that animals are allowed to roam freely, resulting in the deposition of their feces, which in turn contaminates the environment with parasites. This interconnectedness among humans, animals, and the environment was also demonstrated by Sack et al. (2021), where helminth eggs were identified in both animal fecal and soil samples obtained from households in India. Moreover, contact with animals was associated with higher odds of helminth egg contamination in household soil, increasing the risk of zoonotic helminth transmission.

In addition to the presence of human- and animal-parasitic helminths, a significant proportion of nematodes were identified as saprophytic, entomopathogenic, and plant-parasitic. Surveys conducted on soil in crop fields and orchards in Thailand also revealed the presence of entomopathogenic and plant-parasitic nematodes, demonstrating the abundance of various nematode species in the soil environment (Phadungkit et al 2024; Tangchitsomkid and Sontirat 1998; Toida et al. 1996; Vitta et al. 2017). Additionally, the presence of saprophytic nematodes may potentially reduce the abundance of plant pathogens, benefiting soil and crop health (Back et al. 2002; Neher 2010). Nematode diversity in soil has been positively associated with soil health, as these organisms serve as bioindicators of a healthy, robust, and unpolluted soil ecosystem (Laasli et al. 2024). Although the abundance of nematodes was not assessed in this study, the presence of 19 species of nematodes belonging to 13 families in soil samples obtained from livestock farms indicates the potential diversity of helminths within the soil ecosystem.

The importance of utilizing both morphological and molecular methods is emphasized, with *Strongyloides* and *Moniezia* being detectable morphologically but not molecularly. On the other hand, *Fasciola* and *Ascaridia* were detected molecularly but not morphologically. Our results present the first use of both morphological and molecular techniques to identify helminths from soil livestock farms in Thailand, reducing detection bias associated with each technique. The use of molecular methods is beneficial as it allows specific identification of helminths and increased detection sensitivity. However, given the potential for DNA degradation, particularly in environmental samples, molecular assays and primers will have to be sensitive for soil-based detection. This study was limited by utilizing only the nuclear genetic markers (18S rRNA gene and ITS2 region) for molecular identification, whereas the incorporation of a mitochondrial genetic marker might enhance the resolution of species-level detection. In addition, the physical characteristics of the collected soil, which could be useful information relating to the abundance and diversity of helminths in the soil, have not been taken into account.

## Conclusion

By utilizing soil sampling as a non-invasive method for the detection and identification of helminths, this study demonstrated the presence of both human- and animal-parasitic helminths in the soil environment. The soil matrix, serving as a shared environment and reservoir, facilitates zoonotic transmission of helminthic infection. The results emphasize the importance of a One Health approach to mitigate disease transmission. Soil-based detection can serve as a strategy for the surveillance of helminthic infections, allowing for the implementation of appropriate measures, such as deworming or improving sanitation, on livestock farms. Future studies can incorporate soil-based methods for parasitic helminth detection for sustainable control.

## Supplementary information

Below is the link to the electronic supplementary material.

MOESM 1 (DOCX 22.0 KB)

MOESM 2 (DOCX 2.00 MB)

## Data Availability

The dataset supporting the conclusions of this article is included within the article and its supplementary file.
