# Supplementary material for: One Health implications and first evidence of environmental contamination of helminths in soil from goat farms in Ratchaburi, Thailand
Source: Parasitol Res. 2025 Aug 7;124(8):89. doi: 10.1007/s00436-025-08541-w (PMC12331789; doi:10.1007/s00436-025-08541-w)
Supplement: Supplementary file 1 — (DOCX 22.0 KB) [file 436_2025_8541_MOESM1_ESM.docx]

**Supplementary file 1. Sequences used for phylogenetic analysis**

| **Genetic marker** | **Phylum** | **Family** | **Species** | **NCBI accession number** |
| --- | --- | --- | --- | --- |
| 18S | Nematoda | Tylenchidae | *Filenchus misellus* | KJ869308 |
|  |  |  | *Gracilacus* sp. | KR232931 |
|  |  | Allantonematidae | *Howardula* sp. | AF519233 |
|  |  |  | *Howardula aoronymphium* | AF519229 |
|  |  | Cephalobidae | *Pseudacrobeles* sp. | KU180672 |
|  |  |  | *Cervidellus alutus* | AF202152 |
|  |  |  | *Cephalobus cubaensis* | PP182253 |
|  |  | Angunidae | *Ditylenchus* sp. | KJ636300 |
|  |  | Panagrolaimidae | *Panagrolaimus davidi* | AJ567385 |
|  |  |  | *Halicephalobus* sp. | MK087059 |
|  |  | Rhabditidae | *Diploscapter coronatus* | AY593921 |
|  |  |  | *Diploscapter coronatus* | KJ636377 |
|  |  |  | *Protorhabditis* sp. | AF083001 |
|  |  |  | *Rhabditis blumi* | U13935 |
|  |  |  | *Parasitorhabditis obtusa* | EU003189 |
|  |  | Strongylidae | *Oesophagostomum muntiacum* | LC415112 |
|  |  |  | *Oesophagostomum aculeatum* | AB677956 |
|  |  | Trichostrongylidae | *Haemonchus contortus* | EU086374 |
|  |  |  | *Trichostrongylus colubriformis* | AJ920350 |
|  |  | Diplogasteridae | *Oigolaimella* sp. | AB478630 |
|  |  |  | *Diplogastrellus gracilis* | KJ877216 |
|  |  | Neodiplogasteridae | *Mononchoides macrospiculum* | MW007717 |
|  |  |  | *Mononchoides* sp. | KJ877210 |
|  |  | Ascaridiidae | *Ascaridia galli* | EF180058 |
|  |  | Trichuridae | *Trichuris* sp. | MW717993 |
|  |  |  | *Trichuris leporis* | HF586913 |
|  |  | Dorylaimidae | *Mesodorylaimus subtilis* | MG921248 |
|  |  |  | *Mesodorylaimus* sp. | MG921251 |
|  |  |  | *Mesodorylaimus centrocercus* | AY284799 |
|  | Outgroup |  | *Gordionus alpestris* | KT202292 |
|  |  |  | *Nectonema* sp. | LC605988 |
|  | Platyhelminthes (trematode) | Fasciolidae | *Fasciola gigantica* | MN970010 |
|  |  |  | *Fasciola gigantica* | MF077354 |
|  |  |  | *Fasciola gigantica* | ON661089 |
|  |  |  | *Fasciola hepatica* | AJ004969 |
|  |  |  | *Fascioloides magna* | EF534989 |
|  |  | Echinostomatidae | *Echinostoma caproni* | L06567 |
|  |  |  | *Echinostoma revolutum* | AY222132 |
|  |  |  | *Echinostoma paraensei* | FJ380226 |
|  |  | Echinochasmidae | *Echinochasmus milvi* | LT904765 |
|  |  |  | *Echinochasmus japonicus* | LT904764 |
|  |  | Gastrothylacidae | *Gastrothylax crumenifer* | JX518986 |
|  |  |  | *Carmyerius spatiosus* | JX518972 |
|  |  |  | *Fischoederius elongatus* | JX518982 |
|  | Outgroup |  | *Schistosoma mekongi* | AY157228 |
|  | Platyhelminthes (cestode) | Davaineidae | *Raillietina sonini* | EU665468 |
|  |  |  | *Raillietina tunetensis* | EU665465 |
|  |  |  | *Raillietina* sp. | EU665467 |
|  |  |  | *Raillietina mitchelli* | AY382315 |
|  |  |  | *Raillietina dromaius* | AY382314 |
|  |  |  | *Raillietina dromaius* | AY382314 |
|  |  |  | *Raillietina chiltoni* | AY382313 |
|  |  |  | *Raillietina echinobothrida* | MH119095 |
|  |  |  | *Skrjabinia cesticillus* | AY382316 |
|  |  |  | *Fuhrmannetta malakartis* | EU665463 |
|  | Outgroup |  | *Diphyllobothrium latum* | KF218251 |
| ITS2 | Nematoda | Trichostrongylidae | *Haemonchus placei* | AJ577466 |
|  |  |  | *Haemonchus placei* | MH481603 |
|  |  |  | *Haemonchus longistipes* | MK936880 |
|  |  |  | *Haemonchus similis* | KY741886 |
|  |  |  | *Haemonchus contortus* | EU084691 |
|  |  |  | *Haemonchus contortus* | KX534104 |
|  |  |  | *Mecistocirrus digitatus* | AB222060 |
|  |  |  | *Teladorsagia circumcincta* | KC295420 |
|  |  |  | *Teladorsagia trifurcata* | JQ889797 |
|  |  |  | *Marshallagia occidentalis* | AH010211 |
|  |  |  | *Marshallagia marshalli* | MG011724 |
|  |  |  | *Trichostrongylus colubriformis* | KC337070 |
|  |  |  | *Trichostrongylus colubriformis* | JF680985 |
|  |  |  | *Trichostrongylus axei* | KC337066 |
|  |  |  | *Trichostrongylus retortaeformis* | JX046418 |
|  |  |  | *Trichostrongylus vitrinus* | JF680986 |
|  |  |  | *Trichostrongylus vitrinus* | KR020010 |
|  | Outgroup |  | *Trichuris discolor* | AB367795 |
