# Supplementary material for: One Health implications and first evidence of environmental contamination of helminths in soil from goat farms in Ratchaburi, Thailand
Source: Parasitol Res. 2025 Aug 7;124(8):89. doi: 10.1007/s00436-025-08541-w (PMC12331789; doi:10.1007/s00436-025-08541-w)

**Supplementary file 2.** Representative images of helminth eggs identified in the soil samples. Strongylid-like eggs (A to C), *Strongyloides* (D), *Moniezia* (E), and *Trichuris* (F).


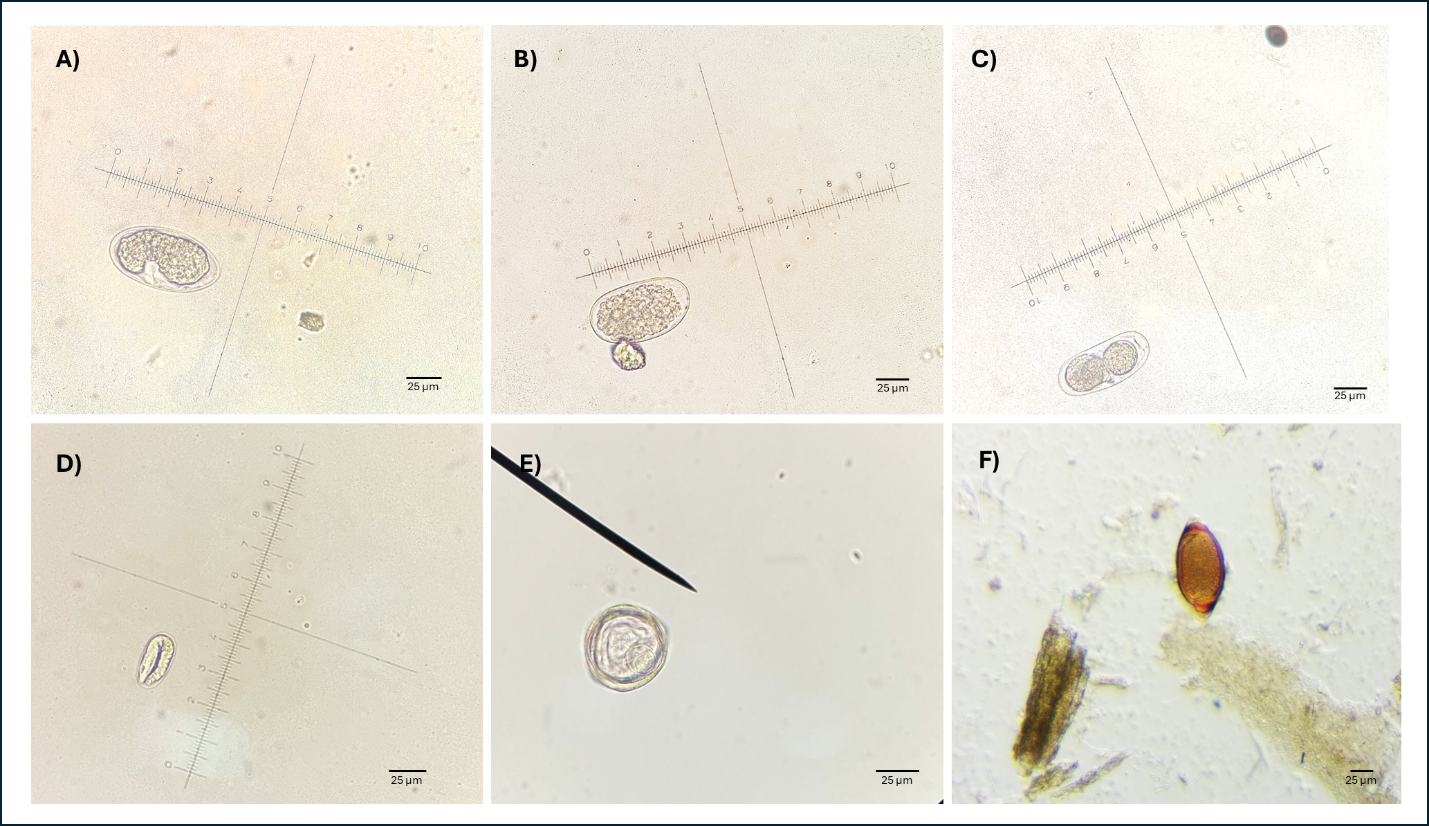

Supplement: Supplementary file 2 — (DOCX 2.00 MB) [file 436_2025_8541_MOESM2_ESM.docx]
